# Impact of sampling site on Activated Clotting Time (ACT) during cryoballoon ablation: Left atrium vs femoral sheath

**DOI:** 10.1016/j.ahjo.2026.100995

**Published:** 2026-10-24

**Authors:** Gilad Margolis, Kirill Berezhnoi, Ofir Goldhaber, Ofer Kobo, Mark Kazatsker, Ma'anit Shapira, Rami Abu-Fanne, Ariel Roguin, Eran Leshem

**Affiliations:** a Technion Israel Institute of Technology, The Ruth and Bruce Rappaport Faculty of Medicine, Technion City, Haifa, Israel; b Division of Cardiovascular Medicine, Hillel Yaffe Medical Center, Hadera, Israel; c Clinical Laboratory Division, Hillel Yaffe Medical Center, Hadera, Israel

**Keywords:** Activated clotting time (ACT), Unfractionated heparin (UFH), Pulmonary vein isolation (PVI), Cryoballoon

## Abstract

**Background:**

Activated clotting time (ACT) is used to monitor unfractionated heparin (UFH) during left atrial (LA) ablation procedures. Inadequate anticoagulation increases thromboembolic risk, while excessive UFH may increase bleeding. ACT can be sampled from a femoral venous sheath (ACT_FV_) or from oxygenated blood within the LA (ACT_LA_). The influence of sampling site on ACT values during atrial fibrillation ablation is unknown.

**Methods:**

In this prospective observational study, consecutive patients undergoing cryoballoon pulmonary vein isolation received intravenous UFH. ACT was sampled sequentially from the LA and from a femoral venous sheath. All samples were analyzed on the same point-of-care ACT machine. Non-inferiority of LA relative to femoral venous sampling was tested against a 30-s margin, with agreement assessed by Cohen's kappa at 300 s and Bland-Altman analysis.

**Results:**

Sixty-two patients were recruited (42% female; CHA_2_DS_2_-VASc 3.1 ± 1.8); 61 had paired samples. Mean ACT_LA_ was 365 ± 83 and ACT_FV_ was 359 ± 65 s (*p* = 0.455). The mean paired difference (ACT_LA_ − ACT_FV_) was +6.1 s (upper one-sided 97.5% confidence limit +22.4 s), below the 30-s non-inferiority margin (*p* = 0.002). Bland-Altman limits of agreement were − 119 to +131 s, with significant proportional bias (β = 0.288, *p* = 0.017). Dichotomized at 300 s, Cohen's kappa was 0.586 (moderate concordance).

**Conclusions:**

ACT sampling from the LA was non-inferior to femoral venous sampling within a 30-s margin, supporting the use of either sampling site during LA ablation. Despite notable variability between paired measurements, unexpected ACT values should prompt repeat testing rather than be attributed to the sampling site.

## 1 Introduction

The activated clotting time (ACT) is routinely used to monitor and titrate anticoagulation therapy with unfractionated heparin (UFH) during atrial fibrillation (AF) ablation procedures. [1] Inadequate anticoagulation during the procedure increases the risk for thrombotic complications, specifically cerebral thromboembolism. Excessive anticoagulation may be associated with an increased risk of bleeding; however, the upper limit of targeted ACT associated with internal bleeding is unknown. [2]

Based on observational data, both American and European position papers recommend a target ACT >300 s during AF ablation. However, the ACT sampling site is not defined and is left to the operator's discretion. [1], [3] Initially validated on venous blood sampling, [4], [5] the ACT test has since been evaluated for differential results by different sampling sites during cardiac procedures [6], [7], [8], [9], [10], [11] and surgeries. [12] While some studies suggested higher ACT values in venous blood compared with arterial sampling, [6], [7] other studies suggested the opposite, [8], [9] or no difference at all. [10] The influence of the sampling site on ACT values has not been investigated in patients undergoing AF ablations.

We aimed to compare ACT values from different sampling sites, namely the venous femoral sheath (ACT_FV_) and oxygenated blood directly from the LA (ACT_LA_), during cryoballoon pulmonary vein isolation (PVI) procedures.

## 2 Methods

### 2.1 Study design and patient population

An investigator-initiated, prospective, single-center, observational study comparing ACT results between different sampling sites during cryoballoon PVI procedures performed by two operators consecutively, between March 2023 and September 2024. This study was conducted in accordance with the principles of the Helsinki Declaration. The local institutional Helsinki (ethics) review committee approved the study protocol (Hillel Yaffe Medical Center Institutional Helsinki Committee, approval number 0001–23-HYMC). Procedural indications were according to published guidelines, [13] including symptomatic, paroxysmal or persistent AF, with most patients being drug refractory. Unless contraindicated, patients began anticoagulation therapy at least one month before to the procedure, which remained uninterrupted during the periprocedural period. All patients provided written informed consent prior to the procedure. No patient was excluded from this analysis.

### 2.2 Procedural workflow

All procedures were carried out under conscious sedation using a combination of midazolam, propofol, and fentanyl. Immediately after ultrasound-guided femoral vein puncture, baseline ACT levels were sampled. Then, all patients were treated with UFH intravenously at a standardized dose of 100 international units (IU) per kg and rounded up (67 kg patient received 7000 IU UFH). UFH was administered exclusively through a peripheral intravenous cannula in the upper limb; no heparin was injected through the femoral venous sheath or through the transseptal sheath before ACT sampling. Following transseptal puncture, a steerable sheath (15.9-F POLARSHEATH™, Boston Scientific or 15-F FlexCath Advance™, Medtronic), flushed with normal saline using a syringe as per manufacturer instructions prior to insertion into the body, was introduced to the LA using an over-the-wire delivery technique. ACT_LA_ levels were then sampled from the LA via the steerable sheath. Importantly, the ACT_LA_ sample was obtained before the sheath was connected to a continuous heparinized saline flush; at the time of sampling, both sheaths had therefore been primed with normal saline alone. The steerable sheath was subsequently connected to a carefully prepared continuous heparinized saline flush. Immediately upon reading the ACT_LA_ results, another ACT sample (ACT_FV_) was obtained from the femoral venous sheath which was prepared in the same manner as the LA sheath ensuring comparable sampling conditions between LA and femoral vein sheaths.

ACT samples from both sheaths were tested on the same point-of-care ACT machine [Actalyke MINI II Activated Clotting Time test system (Helena Laboratories, Beaumont, Texas, USA)] which has a measurement range of 0–1500 s. The system was routinely checked by our lab quality personnel and calibrated according to the manufacturer's instructions. Sampling method in both sampling sites consisted of drawing 10 cc of blood volume (dead space) which was subsequently discarded, followed by 3 cc blood withdrawal for the ACT measurement. Immediately after each sample was drawn, it was promptly run on the designated ACT machine to eliminate any delay between sample acquisition and assay initiation. ACT results and sampling timing were recorded by the electrophysiology lab technician. Throughout the ablation procedure, which was performed as previously described, [14] the ACT was maintained at target levels >300 s using UFH boluses, typically ranging from 2000 to 4000 IU, as needed. If additional ACT samples were required during the procedure, they were obtained exclusively from the femoral vein.

### 2.3 Statistical analysis

Sample size calculations were based on the primary comparison of ACT between paired venous and oxygenated blood samples. A previous study evaluating ACT differences between intravenous lines and arterial catheter sheaths during percutaneous coronary intervention (PCI) reported a mean paired difference of 39 s and a standard deviation of 102 s. [9] Using these estimates, a sample size of 56 patients was calculated to provide 80% power at a two-sided α level of 0.05. To account for potential technical sampling failures, 62 patients were enrolled. Continuous variables were summarized as medians and interquartile ranges (IQRs) or means and SDs, and categorical variables as counts and percentages. Mean ACT comparisons between sampling sites were performed using a paired-sample *t*-test.

A one-sided non-inferiority analysis was performed [15] with the femoral venous sheath sample serving as the reference site and the LA sample as the test measurement. Because the clinical ACT target represents a lower threshold (>300 s), the principal concern is overestimation of ACT by the LA sample, potentially resulting in under-anticoagulation. A non-inferiority margin of 30 s was chosen, as this value is smaller than established sources of ACT measurement variability, including assay drift associated with a one-hour delay in testing (approximately 40 s) [16] and inter-platform differences observed in split-sample comparisons (median difference, 87 s) [17]. Non-inferiority was concluded if the upper bound of the two-sided 95% confidence interval (CI) for the mean paired difference (ACT_LA_ − ACT_FV_) was less than 30 s. Given the observed standard deviation of the paired difference, the study had 95% power to demonstrate non-inferiority at a 30 s margin. To assess the opposite direction of potential bias, a complementary one-sided analysis using the same margin was performed.

Agreement between sampling sites was assessed using Cohen's kappa at an ACT cutoff of 300 s. Kappa values of <0.20, 0.20–0.40, 0.41–0.60, 0.61–0.80, and 0.81–1.0 were considered to show poor, fair, moderate, good and very good agreement, respectively. [18] Additionally, Bland-Altman analysis was performed, with the 95% limits of agreement defined as the mean paired difference ± 1.96 SD. Linear regression was used to assess proportional bias between the two sampling sites. A *P* value <0.05 was considered statistically significant. Analyses were performed using SPSS software version 23 (IBM Corp., Armonk, NY).

## 3 Results

### 3.1 Study participants

A total of 62 patients undergoing cryoballoon PVI were enrolled during the study period. The majority of patients (58%) were men, and the mean age was 67 ± 11 years. Baseline characteristics of the study population are presented in detail in Table 1. The mean CHA_2_DS_2_-VASc score was 3.1 ± 1.8. Four patients had a CHA_2_DS_2_-VASc score of 0. One patient had a left appendage closure device in situ, and was not treated with anticoagulation prior to his procedure. No patient received warfarin treatment, and most patients (84%) received apixaban. A total of 7 patients (11%) were on concomitant antiplatelet therapy. Baseline mean levels of INR, serum creatinine, and platelets were within normal range (Table 1).

**Table 1:** Baseline characteristics.

Variable	***n*** = 62
Age (years)	67 ± 11
Male sex	36 (58%)
Weight (kg)	86 ± 14
Body mass index (kg/m^2^)	29 ± 5
Left ventricular ejection fraction (%)	60 (45–60)
Left atrial area (cm^2^)	20.4 ± 9.8
Atrial arrhythmia type	
Paroxysmal AF	35 (56%)
Persistent AF	27 (44%)
Laboratory values	
PT (*sec*)	11.8 (11.4–12.5)
PTT (sec)	31.0 (28.9–33.9)
INR	1.1 (1.0–1.1)
D-dimer (mg/L)	0.2 (0.1–0.4)
Hemoglobin (g/dL)	12.8 ± 1.8
White blood cells (10^3^/μL)	7.2 (5.8–8.1)
Platelets (10^3^/μL)	209 ± 56
Serum creatinine (mg/dL)	0.9 (0.8–1.1)
CRP (mg/L)	1.9 (0.9–8.0)
CHA₂DS₂-VASc score	
Mean ± SD	3.1 ± 1.8
0	4 (7%)
1	8 (14%)
2	12 (21%)
3	12 (21%)
4	10 (17%)
5	7 (12%)
6	2 (3%)
7	2 (3%)
8	1 (2%)
HAS-BLED score	1.5 ± 1.0
Medical comorbidities	
Hypertension	48 (77%)
Diabetes mellitus	20 (32%)
Smoking	11 (18%)
Chronic kidney disease (eGFR <60 mL/min)	16 (26%)
Peripheral artery disease	1 (2%)
Ischemic heart disease	20 (32%)
Prior CVA	3 (5%)
Medication use	
Anticoagulant therapy	
Apixaban	52 (84%)
Rivaroxaban	6 (10%)
Dabigatran	3 (5%)
Warfarin	0
No anticoagulation	1 (2%)
OAC therapy ≥1 month before ablation	61 (98%)
Antiplatelet therapy	7 (11%)
Antiarrhythmic drugs	
Amiodarone	24 (39%)
Dronedarone	16 (26%)
Class Ic agents	9 (15%)
Beta blockers	47 (76%)
Calcium channel blockers	24 (39%)
ACE inhibitors / ARBs	37 (60%)
Statins	37 (60%)

Values are mean ± SD, median (interquartile range) or n (%).

CHA₂DS₂-VASc score was available in 58 patients.

ACE, angiotensin-converting enzyme; AF, atrial fibrillation; ARB, angiotensin II receptor blocker; CRP, C-reactive protein; CVA, cerebrovascular accident; eGFR, estimated glomerular filtration rate; INR, international normalized ratio; OAC, oral anticoagulation; PT, prothrombin time; PTT, partial thromboplastin time; SD, standard deviation.

### 3.2 Procedural characteristics

Complete PVI was confirmed in all patients. The mean duration of the procedure was 71 ± 18 min, with a mean LA dwell time of 57 ± 18 min. Additional procedural details are provided in Table 2. The mean initial UFH dose administered was 9201 ± 1608 IU, and the total UFH dose was 9911 ± 1986 IU (Table 2).

**Table 2:** Procedural characteristics.

Variable	n = 62
Anticoagulation	
First UFH dose (IU)	9201 ± 1608
Total UFH dose (IU)	9911 ± 1986
Sampling intervals	
UFH administration to LA sample (min)	10 (8–13)
UFH administration to femoral vein sample (min)	18 (15–22)
LA to femoral vein sampling interval (min)	7 (5–10)
Procedure	
Procedure time (min)	71 ± 18
LA dwell time (min)	57 ± 18
Common pulmonary vein	3 (5%)
Electrical cardioversion during procedure	22 (36%)
Concomitant CTI ablation	3 (5%)

Values are mean ± SD, median (interquartile range) or n (%).

ACT_FV_- activated clotting time femoral vein; ACT_LA_ - activated clotting time left atrium; CTI, cavo-tricuspid isthmus; LA, left atrium; UFH, unfractionated heparin.

### 3.3 Comparisons of ACT measurements between different sampling sites

Successful ACT measurements were performed from the LA and the femoral venous sheath in 61 patients. One patient had a technical issue with ACT from the femoral vein. The baseline, pre-heparin administration, mean ACT was 139 ± 19 s. This baseline sample was obtained from the femoral venous sheath alone, since LA access does not exist prior to transseptal puncture. After heparin administration, the mean ACT_LA_ was 365 ± 83 s and the mean ACT_FV_ was 359 ± 65 s (*p* = 0.455, Table 3). The median interval from UFH administration to the ACT_LA_ sample was 10 min (IQR 8–13) and to the ACT_FV_ sample 18 min (IQR 15–22) (Table 2).

**Table 3:** ACT measurements (sec) by sampling site and baseline characteristics.

Variable	ACT_LA_ (sec)	ACT_FV_ (sec)	*p* value
Overall (*n* = 61)	365 ± 83	359 ± 65	0.455
Initial UFH dose			
≥10,000 IU (*n* = 25)	348 ± 66	357 ± 61	0.463
<10,000 IU (*n* = 36)	377 ± 91	361 ± 68	0.142
Oral anticoagulant			
Apixaban (*n* = 51)	366 ± 85	358 ± 65	0.381
Rivaroxaban (*n* = 6)	358 ± 79	364 ± 70	0.831
Dabigatran (*n* = 3)	394 ± 81	386 ± 91	0.727
Body weight			
≥80 kg (*n* = 42)	357 ± 87	359 ± 63	0.826
<80 kg (*n* = 19)	384 ± 72	359 ± 70	0.063
CRP level			
≥2 mg/L (*n* = 29)	350 ± 72	362 ± 61	0.246
<2 mg/L (*n* = 32)	379 ± 90	356 ± 69	0.055
Age			
≥75 years (*n* = 18)	383 ± 80	372 ± 62	0.449
<75 years (*n* = 43)	358 ± 83	354 ± 66	0.695
CHA₂DS₂-VASc score			
≥2 (*n* = 46)	358 ± 75	352 ± 66	0.491
=1 (*n* = 8)	411 ± 87	391 ± 57	0.383
=0 (*n* = 4)	356 ± 81	355 ± 51	0.995
Comorbidities			
Chronic kidney disease (*n* = 15)	347 ± 52	350 ± 59	0.817
Hypertension (*n* = 47)	357 ± 83	353 ± 61	0.638
Diabetes mellitus (*n* = 20)	340 ± 62	339 ± 64	0.938
Smoking (*n* = 11)	356 ± 86	353 ± 64	0.868
Ischemic heart disease (*n* = 19)	362 ± 88	361 ± 69	0.949
Concomitant medications			
Any antiarrhythmic drug (*n* = 48)	371±87	365±63	0.573
Any antiplatelet drug (*n* = 7)	396 ± 99	367 ± 56	0.344
Beta blocker (*n* = 46)	372 ± 87	370 ± 66	0.872
Calcium channel blocker (*n* = 23)	370 ± 81	360 ± 60	0.477
ACE inhibitor / ARB (*n* = 36)	378 ± 87	362 ± 62	0.164
Statin (n = 36)	378 ± 78	363 ± 55	0.174

Values are mean ± SD. *p* values are from paired-sample *t*-tests.

CHA₂DS₂-VASc score was unavailable in 3 of the 61 patients with paired samples.

ACE, angiotensin-converting enzyme; ACT_FV_- activated clotting time femoral vein; ACT_LA_ - activated clotting time left atrium; ARB, angiotensin II receptor blocker; CRP, C-reactive protein; SD, standard deviation; UFH, unfractionated heparin.

The mean paired difference (ACT_LA_ − ACT_FV_) was 6.1 s (SD 63.7 s), with a two-sided 95% CI ranging from −10.2 to 22.4 s. Because the upper confidence bound (22.4 s) was below the non-inferiority margin of 30 s, non-inferiority of the LA measurement relative to the femoral venous sheath reference was demonstrated (one-sided *p* = 0.002). In the complementary analysis assessing the opposite direction of bias, the lower confidence bound (−10.2 s) remained above the margin of −30 s, excluding underestimation of 30 s or greater by the LA sample (one-sided *p* < 0.001).

In subgroup analyses by initial UFH dose, CRP levels, age, CHA_2_DS_2_-VASc score, baseline comorbidities, and medications including DOAC type, no difference between ACT levels by sampling site was observed (Table 3). Patients weighing less than 80 kg had a trend towards lower ACT results from venous testing (*p* = 0.063, Table 3). Subgroup analysis performed only on patients prescribed apixaban, showed no difference in ACT levels between sampling sites, except among those with CRP levels <2 mg/L, in whom the mean ACT_LA_ was 384 ± 93 s and the mean ACT_FV_ was 351 ± 67 s (*p* = 0.022, Supplemental Table 1).

Disagreement in ACT results between sampling sites with clinical significance, where the LA sampling site showed ACT above 300 s and the femoral vein sampling site showed ACT below 300 s, or vice-versa, was observed in 8 patients (13%) (Fig. 1). The Cohen's kappa value was 0.586, indicating moderate concordance for both sampling sites at an ACT cutoff of 300 s (Fig. 1, supplemental Table 2). Bland-Altman analysis showed a mean bias of +6.1 s with 95% limits of agreement from −119 to +131 s, and regression of the paired difference on the mean of the two measurements demonstrated statistically significant proportional bias between the sampling sites (β = 0.288, *r* = 0.31, *p* = 0.017), mainly at higher ACT values (>400 s, Fig. 2).

**Fig. 1:**
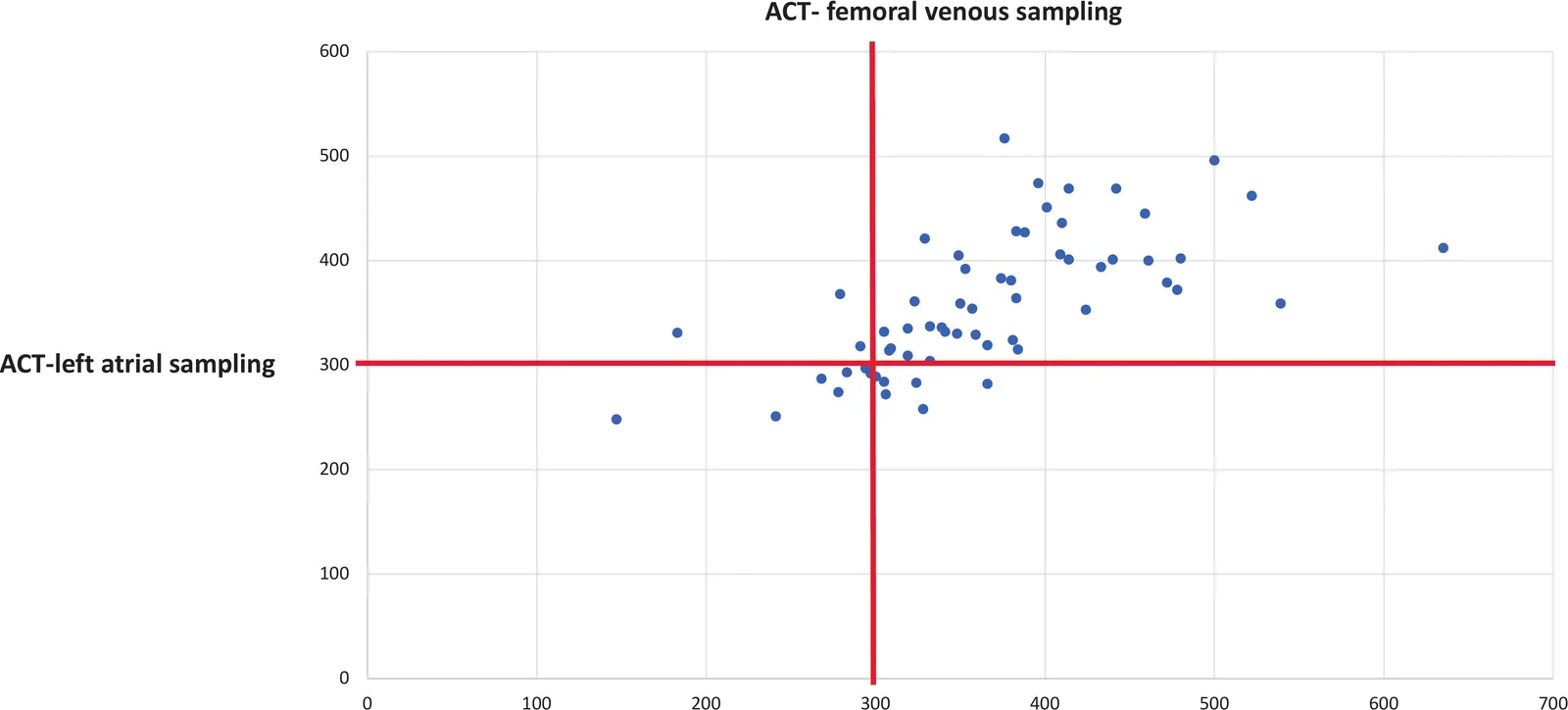
Distribution of ACT values: venous femoral vs left atrial sampling. ACT values in both x and y axes are in seconds. ACT- activated clotting time.

**Fig. 2:**
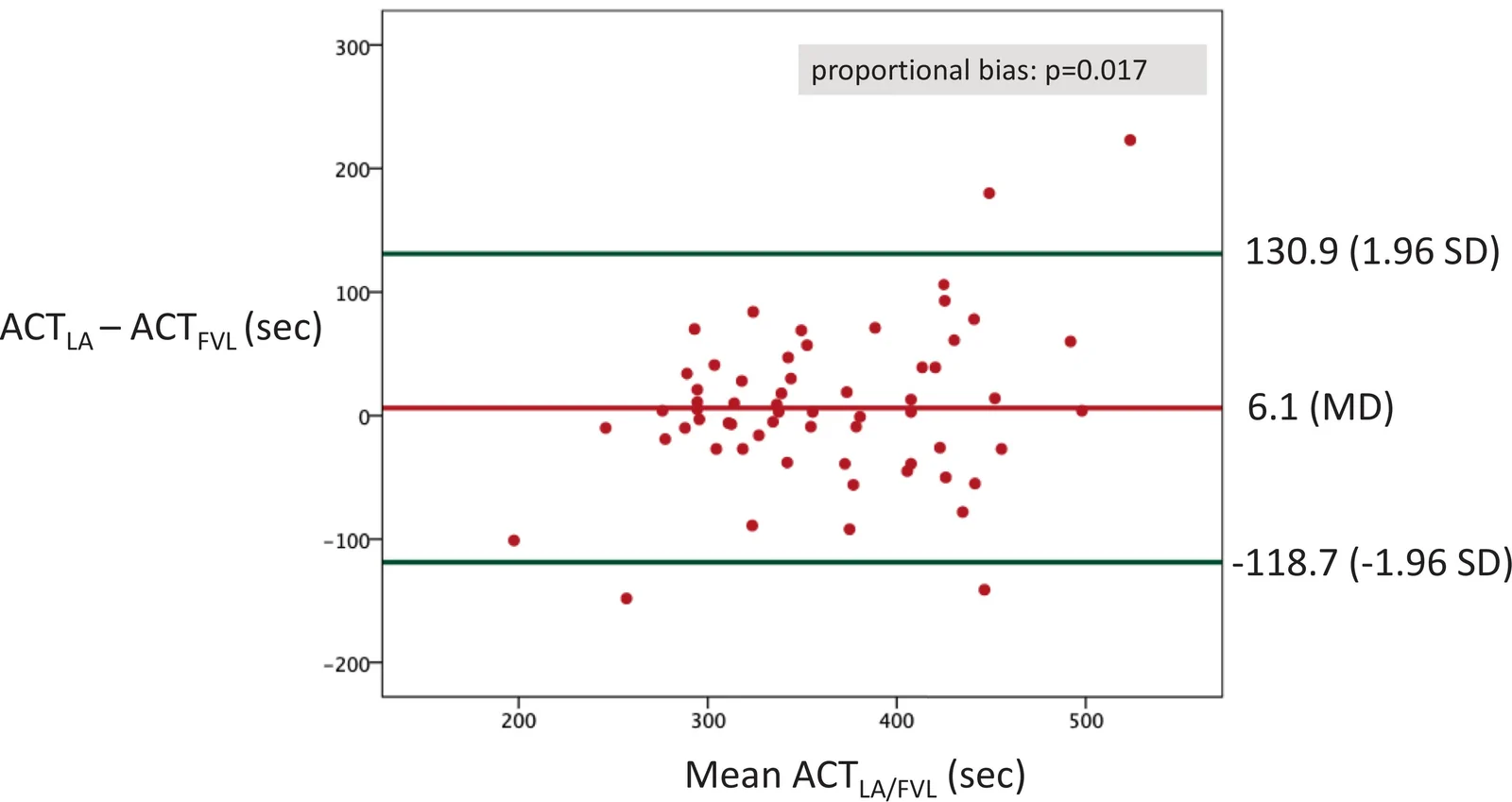
Bland-Altman plot of ACT_LA_ and ACT_FV_ values comparison. ACT_FV_- activated clotting time femoral vein; ACT_LA_ - activated clotting time left atrium; MD- mean difference; SD- standard deviation.

## 4 Discussion

In this study, ACT measurements obtained from the LA were non-inferior to those obtained from the femoral venous sheath during cryoballoon PVI ablations. Although concordance was only moderate when ACT values were dichotomized using the 300-s therapeutic threshold, the mean difference between sampling sites was small and below the non-inferiority margin for clinical relevance. Collectively, these findings suggest that ACT measurements are largely unaffected by whether blood is sampled from the femoral venous sheath or directly from the oxygenated LA circulation following transseptal access.

The impact of the ACT sampling site on ACT measurements during AF ablations has not been investigated. Studies assessing differential ACT results by venous or arterial sampling sites in patients undergoing PCI have reported conflicting results. In two earlier studies, ACT levels were higher in venous compared with arterial sampling, [6], [7] while in two other studies, [8], [9] the opposite was observed. However, these studies utilized different methodologies. In one of the studies, arterial blood samples were drawn from an arterial line that was continuously flushed with heparin. [8] In the other studies, arterial blood was sampled from a normal saline-flushed arterial line. [6], [7], [9] In addition, different ACT measuring machines and ACT measuring timings were employed: a single sample at the PCI ending, [6], [9] or serial ACT measurements during the procedure. [7], [8] The different ACT sampling and measurement protocols that were used may explain the discrepancies in the results. Notably, although not powered for this purpose, no difference in complications rates was observed between venous and arterial ACT guided patients. [7], [8]

In our study, ACT measurements obtained from the LA were non-inferior to those obtained from the femoral venous sheath within a margin of 30 s, supporting the use of either sampling site for protocol development and cohort-level analyses. The mean difference between sites was small and centered near zero, and no significant differences were observed across most subgroups. The only exception was a subgroup of patients receiving apixaban with CRP levels <2 mg/L, in whom mean ACT values were higher in LA samples than in femoral venous samples. Given the multiple subgroup comparisons performed and the absence of a biologically plausible mechanism, the clinical significance of this isolated finding is uncertain and may represent a chance observation.

Although the overall agreement between sampling sites was favorable, agreement at the level of individual measurements was more modest. The 95% limits of agreement ranged from −119 to +131 s, and 8 of 61 patients (13%) were classified differently relative to the clinically relevant threshold of 300 s. Similarly, categorical agreement was only moderate (Cohen's κ = 0.586). Importantly, this variability is unlikely to reflect a systematic effect of sampling location, as evidenced by the negligible mean bias between sites. Rather, it likely reflects the inherent imprecision and within-patient variability of ACT testing. Consistent with this interpretation, Leyvi et al. reported substantial variability in repeated ACT measurements obtained during cardiopulmonary bypass, with discrepancies reaching several hundred seconds. [12] Clinically, these findings suggest that an unexpected or discordant ACT result should prompt repeat testing rather than be attributed to the site from which the sample was obtained.

The Bland-Altman analysis further demonstrated evidence of proportional bias at higher ACT values (>400 s), meaning that when obtaining an increased ACT result, the potential for difference between sampling sites is higher. This analysis is in line with previous research: Heemelaar et al. [9] reported larger ACT deviations between arterial and venous measurements at higher ACT values among PCI-treated patients. Similarly, in a study targeting a lower ACT of 200 s and above [10] no significant difference between venous and arterial ACT values was found among pediatric patients undergoing cardiac catheterizations for congenital heart disease when the mean observed values were ~ 230 s. Taken together, these data suggest that ACT measurements derived from different sampling sites are largely interchangeable within the range most relevant to clinical decision-making, but that variability increases as ACT values become markedly elevated.

From a practical standpoint, in single-shot cryoballoon AF ablation procedures, and potentially for pulse field ablation procedures using similar LA sheaths, our findings suggest that operators may use a blood sample from the LA for ACT measurement immediately after transseptal puncture to streamline workflow. Additional ACT sampling required during these procedures should be performed via the femoral vein to avoid the risk of air embolism associated with unnecessary LA sheath manipulation. Our findings may also extend to radiofrequency AF ablation procedures. During such procedures, blood aspirated from the left atrial sheath after catheter exchange (e.g., swapping a mapping catheter for an ablation catheter) to prevent air introduction can be utilized for ACT sampling, although this approach should be formally validated.

A broader question is whether intraprocedural ACT monitoring itself is evidence-based. The guideline-recommended threshold of >300 s is based largely on observational data, [19] and lower ACT targets have also been reported as safe. [20], [21] Furthermore, ACT values may vary substantially between point-of-care platforms. [22] Accordingly, the present study should not be interpreted as endorsing any specific ACT threshold. Rather, it suggests that, when ACT monitoring is performed, sampling site does not introduce clinically meaningful systematic bias within a single assay platform.

Several limitations warrant consideration. First, the study was conducted solely at a single medical center. Consequently, the generalizability of the findings to other healthcare facilities may be constrained. For example, we performed all procedures under conscious sedation, and did not utilize arterial line for hemodynamic monitoring. Therefore, a commonly used sampling site of oxygenated blood for ACT, such as a radial arterial line, was not used. Second, no patient received background warfarin, 9 patients (15%) were treated with rivaroxaban or dabigatran and only 7 patients (11%) received antiplatelet therapy. Therefore, the findings of this study may not apply to patients treated with these medications. Third, ACT samples were drawn and analyzed sequentially in the same ACT machine and not in parallel. This time lag may potentially introduce inconsistencies to the ACT measurements and time-dependent bias. However, the second ACT test was done immediately after the first ACT test result, leading to an average lag time of around 7 min after first ACT blood withdrawal (Table 2). According to Philip et al. [16], the expected difference in ACT after one hour is approximately 40 s. Consequently, the time delay in our sequential ACT measurements is likely to have a negligible impact on ACT values (at most 4–7 s). However, Philip et al. reported significantly lower mean ACT values (~127 s) compared to our study, which may limit the applicability of their findings to our results. To minimize instrumentation bias, we opted for sequential ACT sampling with a single ACT machine rather than simultaneous measurements with multiple machines, in an effort to ensure consistent results. Fourth, paired baseline measurements before heparin administration could not be obtained because LA access is established only after transseptal puncture; therefore, a site-specific difference independent of anticoagulation cannot be excluded. Of note, Leyvi et al. observed significantly higher ACT values in venous than in arterial samples at baseline during cardiac surgery, a difference that was no longer detectable after heparin administration. [12] Fifth, the choice of the non-inferiority margin may represent a potential limitation. Although the margin was based on clinical considerations and published literature, non-inferiority would not have been demonstrated had a more stringent margin been selected. Nevertheless, the clinical relevance of this difference may be limited. In our cohort, UFH increased ACT by approximately 24 s per 1000 IU, such that the smallest heparin bolus routinely used in practice (2000 IU) would be expected to increase ACT by approximately 48 s. Consequently, a between-site difference of 30 s is smaller than the effect of the smallest routine dose adjustment and would be unlikely, in isolation, to influence clinical management.

In conclusion, ACT measurements obtained from the LA were non-inferior to those obtained from the femoral venous sheath during cryoballoon PVI. The small mean difference between sampling sites supports the use of either site for ACT-guided anticoagulation management. However, variability between paired measurements was notable, suggesting that unexpected ACT values should prompt repeat testing rather than be attributed to the sampling site.

## CRediT authorship contribution statement

**Gilad Margolis:** Writing – review & editing, Writing – original draft, Supervision, Methodology, Formal analysis, Data curation, Conceptualization. **Kirill Berezhnoi:** Writing – original draft, Formal analysis, Data curation. **Ofir Goldhaber:** Methodology, Formal analysis. **Ofer Kobo:** Visualization, Validation, Supervision. **Mark Kazatsker:** Writing – review & editing, Validation, Supervision. **Ma'anit Shapira:** Resources. **Rami Abu-Fanne:** Validation, Supervision. **Ariel Roguin:** Writing – review & editing, Validation, Supervision. **Eran Leshem:** Writing – review & editing, Validation, Supervision, Investigation, Conceptualization.

## Declaration of Generative AI and AI-assisted technologies in the writing process

During the preparation of this work, the authors used Claude (Anthropic) to assist with language editing and drafting. The authors reviewed and edited the output as needed and take full responsibility for the content of the published article.

## Source of funding

None.

## Declaration of competing interest

None.
