# Supplementary material for: Impact of sampling site on Activated Clotting Time (ACT) during cryoballoon ablation: Left atrium vs femoral sheath
Source: Am Heart J Plus. 2026 Oct 24;72:100995. doi: 10.1016/j.ahjo.2026.100995 (PMC13735004; doi:10.1016/j.ahjo.2026.100995)
Supplement: Supplementary file 1 [file mmc1.docx]

**Supplemental Table 1. ACT measurements (sec) by sampling site and baseline characteristics in patients prescribed apixaban**

| **Variable** | **ACT_LA_ (sec)** | **ACT_FV_ (sec)** | **p value** |
| --- | --- | --- | --- |
| Overall (n = 51) | 366 ± 85 | 358 ± 65 | 0.381 |
| **Initial UFH dose** |  |  |  |
| ≥10,000 IU (n = 20) | 338 ± 62 | 345 ± 56 | 0.614 |
| <10,000 IU (n = 31) | 384 ± 94 | 367 ± 69 | 0.176 |
| **Body weight** |  |  |  |
| ≥80 kg (n = 36) | 353 ± 90 | 353 ± 63 | 0.992 |
| <80 kg (n = 15) | 398 ± 65 | 371 ± 70 | 0.101 |
| **CRP level** |  |  |  |
| ≥2 mg/L (n = 27) | 350 ± 75 | 364 ± 64 | 0.233 |
| <2 mg/L (n = 24) | 384 ± 93 | 351 ± 67 | 0.022 |
| **Age** |  |  |  |
| ≥75 years (n = 16) | 383 ± 84 | 373 ± 65 | 0.582 |
| <75 years (n = 35) | 359 ± 86 | 351 ± 65 | 0.505 |
| **CHA₂DS₂-VASc score** |  |  |  |
| ≥2 (n = 39) | 361 ± 77 | 354 ± 67 | 0.437 |
| = 1 (n = 7) | 407 ± 93 | 380 ± 52 | 0.301 |
| **Comorbidities** |  |  |  |
| Chronic kidney disease (n = 12) | 342 ± 56 | 342 ± 59 | 0.991 |
| Hypertension (n = 40) | 355 ± 86 | 352 ± 64 | 0.780 |
| Diabetes mellitus (n = 19) | 335 ± 60 | 336 ± 65 | 0.926 |
| Smoking (n = 8) | 345 ± 73 | 349 ± 50 | 0.870 |
| Ischemic heart disease (n = 15) | 363 ± 94 | 360 ± 71 | 0.871 |
| **Concomitant medications** |  |  |  |
| Any antiarrhythmic drug (n = 42) | 371 ± 88 | 362 ± 63 | 0.437 |
| Any antiplatelet drug (n = 5) | 433 ± 93 | 390 ± 48 | 0.313 |
| Beta blocker (n = 38) | 372 ± 90 | 368 ± 67 | 0.747 |
| Calcium channel blocker (n = 17) | 370 ± 86 | 366 ± 67 | 0.781 |
| ACE inhibitor / ARB (n = 31) | 376 ± 91 | 359 ± 64 | 0.191 |
| Statin (n = 30) | 384 ± 82 | 362 ± 54 | 0.090 |

Values are mean ± SD. p values are from paired-sample t-tests. Only two apixaban-treated patients had a CHA₂DS₂-VASc score of 0; this subgroup is therefore not reported. ACE, angiotensin-converting enzyme; ACT, activated clotting time; ACTFV, ACT sampled from the femoral vein; ACTLA, ACT sampled from the left atrium; ARB, angiotensin II receptor blocker; CRP, C-reactive protein; SD, standard deviation; UFH, unfractionated heparin.

**Supplemental Table 2. Characteristics of the 8 patients with discordant ACT values across the 300 sec threshold**

| **Variable** | **n = 8** |
| --- | --- |
| Age (years) | 63 (61–69) |
| Male sex | 6 (75%) |
| Weight (kg) | 93 (78–101) |
| Body mass index (kg/m²) | 29 (25–33) |
| Left ventricular ejection fraction (%) | 45 (30–60) |
| Left atrial area (cm²) | 26 (24–29) |
| **Atrial arrhythmia type** |  |
| Paroxysmal AF | 4 (50%) |
| Persistent AF | 4 (50%) |
| **Laboratory values** |  |
| PT (sec) | 11.7 (11.2–11.8) |
| PTT (sec) | 26.5 (24.7–30.7) |
| INR | 1.0 (1.0–1.1) |
| D-dimer (mg/L) | 0.3 (0.2–0.8) |
| Hemoglobin (g/dL) | 13.0 (12.4–13.5) |
| White blood cells (10³/µL) | 6.9 (5.9–7.6) |
| Platelets (10³/µL) | 178 (118–277) |
| Serum creatinine (mg/dL) | 0.9 (0.8–1.3) |
| CRP (mg/L) | 1.4 (0.8–5.5) |
| **Medical comorbidities** |  |
| Hypertension | 7 (88%) |
| Diabetes mellitus | 5 (63%) |
| Smoking | 2 (25%) |
| Chronic kidney disease (eGFR <60 mL/min) | 2 (25%) |
| Peripheral artery disease | 0 |
| Ischemic heart disease | 2 (25%) |
| Prior CVA | 0 |
| **Medication use** |  |
| Apixaban | 8 (100%) |
| Rivaroxaban | 0 |
| Dabigatran | 0 |
| Warfarin | 0 |
| OAC therapy ≥1 month before ablation | 8 (100%) |
| Antiplatelet therapy | 0 |
| Antiarrhythmic drugs |  |
| Amiodarone | 3 (38%) |
| Dronedarone | 2 (25%) |
| Class Ic agents | 2 (25%) |
| Beta blockers | 7 (88%) |
| Calcium channel blockers | 2 (25%) |
| ACE inhibitors / ARBs | 6 (75%) |
| Statins | 5 (63%) |

Values are median (interquartile range) or n (%). Discordance was defined as an ACT >300 sec at one sampling site and ≤300 sec at the other. ACE, angiotensin-converting enzyme; ACT, activated clotting time; AF, atrial fibrillation; ARB, angiotensin II receptor blocker; CRP, C-reactive protein; CVA, cerebrovascular accident; eGFR, estimated glomerular filtration rate; INR, international normalized ratio; OAC, oral anticoagulation; PT, prothrombin time; PTT, partial thromboplastin time.
